# An Autumn Campaign

**Published:** 1920-09-25

**Authors:** 


### THE RED CROSS PEACE PROGRAMME.

### An Autumn Campaign.

This autumn is to see a special effort extending over the two months of October and November in aid of the voluntary hospitals. The campaign will have two forms, with a day devoted in each month to the culmination of the propaganda. The occasion is perhaps of even greater interest to the London hospitals than to those in the provinces, not only because the difficulties of the London hospitals are more acute, but because the problem in London is to raise large sums by very small contributions, and no individual hospital in London seems capable at present of organising successfully this source of income. The consequence is that the attempt devolves upon the Red Cross, which hopes to continue to gather the support of all and sundry which it collected so successfully during the war. Just as the Sunday and Saturday Funds appeal primarily to special classes of the community, so the autumn campaign will appeal to the pockets of the general public. A good deal will depend upon the success of the effort, because the days are passed when the Red Cross, merely by a letter to the newspapers, could have all the money which it wanted for the asking. How, then, is the organisation to be directed?

### The Scheme in Outline.

The familiar device of "Our Day" is the first part of the programme, and the date of this occasion has been fixed for Thursday, October 14, because that is the nearest available date to the anniversary of the day on which the two societies joined forces to aid the wounded in the war. But "Our Day," October 14, will be the culmination of a week's effort. The details of the programme have not been made public, but presumably no means of propaganda will be left untried. Those who favour a central appeal in place of different appeals from the separate hospitals have here an opportunity, for propaganda in London will have to be arranged not only from the centre, but in the separate districts, and in these the hospitals might arrange with the Joint Council of the Red Cross to help. For though the Joint Council has many objects to help, the voluntary hospitals are chief among them.

The Council's prime endeavours are to raise one million a year; to maintain the orthopædic centres for pensioners; to aid the Central Council for Infant and Child Welfare (and there are now 1,718 centres and 1,564 health visitors); to assist many sanatoria in which the Joint Council is interested; to extend the service of 300 motor-ambulances already established; and last, in a category of its own, to assist the voluntary hospitals. The recent survey by Sir Napier Burnett was concerned with the provincial hospitals, but the plan for buying stores in bulk is one which may prove of value to the London institutions. The Red Cross already possesses large stores for the reception of non-perishable goods, and if these can be bought in the country of origin not only will retail profits be saved, but to some extent those of the wholesalers; and in so far as prices are forced upwards by the competition of different hospitals in the open market, a further saving would be made. Besides this, a Central Fund is required, an object which should commend itself to the richer donors and those with money to bequeath to charity. The work parties and Red Cross workrooms are also to be continued, so that a kind of national "linen league" may be formed, affiliated to the Central Workrooms of the Joint Societies, now open at 16 West Halkin Street, S.W. 1. Voluntary helpers are wanted for these work parties, and funds are needed to buy the materials.

### The County Committees.

We trust that the Red Cross County Committees, of which we have already published some account, will prove their value during this week in October. From October 9 to 13 strenuous efforts are planned that "Our Day" on the 14th may be the success for which we all hope. Since the County Committees will also co-operate in the distribution of the money, they and the local hospitals have every motive to assist each other. For if the impulse is sufficient and the organisation thorough "Our Day" will become an annual event. Since the money collected in each county will be distributed there our eyes will watch with the keenest interest the fortunes of the effort in London. We live in days when large sums are raised by innumerable pennies, and since the London hospitals, as we have pointed out, do not make much of this branch of their finance, the success of the Red Cross "Our Day" collection becomes of unusual importance.

The second form of the effort is to be made with the help of the churches, and Sunday, November 14, has been set apart for collections throughout the kingdom the nearest Sunday to Armistice Day. The Archbishops, Cardinal Bourne, and the heads of all the Free Churches have generously issued a joint appeal urging this collection upon the clergy. It is a generous act, for the clergy have many claims upon the offertories, but what may be called Armistice Sunday should appeal to the sympathy and gratitude of all. The Imperial War Relief Fund, which is concerned with the disastrous prevalence of typhus in Central Europe, will share in the Sunday collection.

The million which the Joint Council hopes to raise is really required by the provincial hospitals. The London hospitals are a separate problem; in a sense they are the real problem. The Red Cross effort in London is in the nature of a joint appeal for their benefit.

An arrangement has been made between the League of Mercy, the Hospital Saturday Fund in London, and the Joint Council, under which the workers of the League of Mercy and of the Hospital Saturday Fund will assist in the appeal to be made during Red Cross Week.

